# Supplementary material for: Exploring whole-body kinematics when eating real foods with the dominant hand in healthy adults
Source: PLoS One. 2021 Oct 28;16(10):e0259184. doi: 10.1371/journal.pone.0259184 (PMC8553040; doi:10.1371/journal.pone.0259184)
Supplement: Attachment — Submitted filename: Response to Reviewers.docx [file pone.0259184.s009.docx]

October 13, 2021

Bernadette Ann Murphy

Academic Editor

*PLoS ONE*

Dear Dr. Murphy:

**Re.: # PONE-D-20-39538R1**

**Title: Exploring whole-body kinematics when eating real foods with the dominant hand in healthy adults**

Thank you for your email containing your and the reviewer’s comments regarding our manuscript. The reviewers’ comments were very helpful for revising and improving our manuscript, as well as for offering important guidance for our research.

The manuscript has been carefully rechecked, and the necessary changes have been made in accordance with your suggestions. As such, all revisions have been clearly highlighted in the manuscript with track changes. The main corrections and point-by-point responses to the reviewers’ comments are provided below.

Our funding statement is as follows: This study was partly funded by a grant from the University of Miyazaki Hospital. The funder had no role in the preparation of the manuscript. There was no additional external funding received for this study.

We hope that our responses and revisions have adequately addressed the reviewers’ concerns, and that the revised manuscript will now meet the high standards required for publication in your esteemed journal. We look forward to hearing from you.

Yours sincerely,

Jun Nakatake

Rehabilitation Unit, University of Miyazaki Hospital

Kiyotake-cho Kihara 5200, Miyazaki-city, Miyazaki, 889-1692, Japan

Phone: +81-985-85-9849

E-mail: [jyun_nakatake@med.miyazaki-u.ac.jp](mailto:jyun_nakatake@med.miyazaki-u.ac.jp)

**Manuscript ID: PONE-D-20-39538R1**

**Title: Exploring whole-body kinematics when eating real foods with the dominant hand in healthy adults**

Point-by-Point Response

Thank you for reviewing our manuscript and providing thoughtful comments. We have revised our manuscript accordingly. Please note that the changes made do not influence the content, conclusions, or framework of the paper. We have not listed below all minor changes made; however, these can be viewed as “tracked changes” in the revised manuscript.

**Reviewer #1: Critique and Responses**

Comment: There is still limited justification for the comparison between the four different phases - it does not seem necessary and it is not clear what added value is gained from doing this. I think the presentation of movement patterns and ranges of motion in the different phases is what is important here and I was glad to see the new figures that replaced the tables. Nevertheless, the comparison between the different phases have been considerably watered down in this version and are not made to be the focus which is good in my opinion.

**Response:** The following underlined parts were added to the Discussion section.

“The different movements from this pattern, especially the reaching phase, and the mean angles of the shoulder internal rotation and the wrist flexion were the smallest. The spooning phase also required large ROMs of forearm pronation (decreasing angles) as well as of wrist flexion (increasing and decreasing angles) and radial deviation (decreasing angles). Knowledge of the movement patterns that we have described may be useful for the comprehensive assessment of eating and optimization of upper and lower limb, neck, and trunk movements during eating. To facilitate or compensate for the normal positions and movements for each eating phase, appropriate positioning, seating equipment, upper extremity and posture control, and orthotics [3, 4] referring to the mean angles, the ROMs, and the changes in joint angles of the present study could be provided in practice.” (page 18)

“Maximal shoulder flexion and abduction angles in individuals with rotator cuff impingement are approximately the same as those in healthy individuals [7]; thus, these two groups’ shoulder movements would not be different in the mouth phase examined here. The severe upper extremity function in children with unilateral cerebral palsy and their maximal forearm pronation angle during eating are positively correlated [9], which could be observed in our spooning phase.” (page 20)

Comment: The sentence describing the instructions given to the participants is quite complex, was this the language used to describe this? Or does it sound complex because it is a literal translation of the instructions?

**Response:** The following were the approximate instructions provided to the participants. Underlined sections underwent minor revisions.

“Participants held a spoon with their right hand and received the task instruction, “please eat three spoons full of yogurt without a break, using habitual whole-body movements and speed, not looking away, and with your left hand resting on your left thigh.” (page 7)
